# Treatment eligibility and retention in clinical HIV care: A regression discontinuity study in South Africa

**DOI:** 10.1371/journal.pmed.1002463

**Published:** 2017-11-28

**Authors:** Jacob Bor, Matthew P. Fox, Sydney Rosen, Atheendar Venkataramani, Frank Tanser, Deenan Pillay, Till Bärnighausen

**Affiliations:** 1 Department of Global Health, Boston University School of Public Health, Boston, Massachusetts, United States of America; 2 Department of Epidemiology, Boston University School of Public Health, Boston, Massachusetts, United States of America; 3 Africa Health Research Institute, Somkhele, South Africa; 4 Health Economics and Epidemiology Research Office, Department of Internal Medicine, School of Clinical Medicine, Faculty of Health Sciences, University of Witwatersrand, Johannesburg, South Africa; 5 Department of Medical Ethics and Health Policy, Perelman School of Medicine, University of Pennsylvania, Philadelphia, Pennsylvania, United States of America; 6 School of Nursing and Public Health, University of KwaZulu-Natal, Durban, South Africa; 7 Centre for the AIDS Programme of Research in South Africa (CAPRISA), University of KwaZulu-Natal, South Africa; 8 Research Department of Infection & Population Health, University College London, London, United Kingdom; 9 Department of Virology, University College London, London, United Kingdom; 10 Department of Global Health and Population, Harvard T.H. Chan School of Public Health, Boston, Massachusetts, United States of America; 11 Institute of Public Health, University of Heidelberg, Heidelberg, Germany; Makerere University College of Health Sciences, School of Public Health, UGANDA

## Abstract

**Background:**

Loss to follow-up is high among HIV patients not yet receiving antiretroviral therapy (ART). Clinical trials have demonstrated the clinical efficacy of early ART; however, these trials may miss an important real-world consequence of providing ART at diagnosis: its impact on retention in care.

**Methods and findings:**

We examined the effect of immediate (versus deferred) ART on retention in care using a regression discontinuity design. The analysis included all patients (*N* = 11,306) entering clinical HIV care with a first CD4 count between 12 August 2011 and 31 December 2012 in a public-sector HIV care and treatment program in rural South Africa. Patients were assigned to immediate versus deferred ART eligibility, as determined by a CD4 count < 350 cells/μl, per South African national guidelines. Patients referred to pre-ART care were instructed to return every 6 months for CD4 monitoring. Patients initiated on ART were instructed to return at 6 and 12 months post-initiation and annually thereafter for CD4 and viral load monitoring. We assessed retention in HIV care at 12 months, as measured by the presence of a clinic visit, lab test, or ART initiation 6 to 18 months after initial CD4 test. Differences in retention between patients presenting with CD4 counts just above versus just below the 350-cells/μl threshold were estimated using local linear regression models with a data-driven bandwidth and with the algorithm for selecting the bandwidth chosen ex ante. Among patients with CD4 counts close to the 350-cells/μl threshold, having an ART-eligible CD4 count (<350 cells/μl) was associated with higher 12-month retention than not having an ART-eligible CD4 count (50% versus 32%), an intention-to-treat risk difference of 18 percentage points (95% CI 11 to 23; *p <* 0.001). The decision to start ART was determined by CD4 count for one in four patients (25%) presenting close to the eligibility threshold (95% CI 20% to 31%; *p <* 0.001). In this subpopulation, having an ART-eligible CD4 count was associated with higher 12-month retention than not having an ART-eligible CD4 count (91% versus 21%), a complier causal risk difference of 70 percentage points (95% CI 42 to 98; *p <* 0.001). The major limitations of the study are the potential for limited generalizability, the potential for outcome misclassification, and the absence of data on longer-term health outcomes.

**Conclusions:**

Patients who were eligible for immediate ART had dramatically higher retention in HIV care than patients who just missed the CD4-count eligibility cutoff. The clinical and population health benefits of offering immediate ART regardless of CD4 count may be larger than suggested by clinical trials.

## Abstract 2

Jacob Bor and colleagues use data on retention in care for people with HIV whose CD4 counts are just above or just below cutoffs for ART initiation to estimate real-world benefit of expanding ART eligibility.

## Author summary

**Why was this study done?:**

- Starting antiretroviral therapy (ART) at HIV diagnosis has health benefits relative to deferring therapy.
- Understanding the magnitude of these benefits is important for countries expanding treatment eligibility.
- Clinical trials may underestimate the real-world benefits of immediate ART, because in such trials, patients assigned to deferred ART are retained in care and carefully monitored to determine future ART eligibility and to assess trial endpoints.
- Although clinical trials have shown the biological benefits of early ART, the behavioral impacts on retention in care are unknown.

**What did the researchers do and find?:**

- We assessed the association between immediate versus deferred ART eligibility and clinical retention among 11,036 patients presenting for HIV care in rural South Africa, the country with the largest HIV epidemic and largest ART program worldwide.
- Using a regression discontinuity design, we compared 12-month retention in care among patients presenting just above versus just below the 350-cells/μl CD4 threshold used to determine ART eligibility during the period of study.
- We found that, among these patients, immediate eligibility increased initiation of ART by 25 percentage points and 12-month retention by 18 percentage points.
- Among patients starting ART because they had an eligible CD4 count, 12-month retention was 91%; among patients prevented from starting ART because their CD4 count was above 350 cells/μl, 12-month retention was 21%.

**What do these findings mean?:**

- Offering ART immediately at diagnosis improves retention in care, reducing the number of patients who screen positive for HIV but are then lost to follow-up before they can start ART.
- The benefits of offering ART immediately at diagnosis, regardless of CD4 count, may be greater than previously thought.

## Introduction

Mass provision of HIV treatment has improved life expectancy in southern Africa [1–3], yet HIV remains the leading cause of death and disability [4]. Recent clinical trials show health benefits to antiretroviral therapy (ART) at high CD4 counts [5–7]; WHO now recommends starting HIV patients on ART at diagnosis [8], and many countries have moved to “treat all” policies [9].

Although clinical efficacy has been demonstrated under trial conditions, the effect of immediate versus deferred ART in clinical settings in the “real world” is largely unknown. In addition to the direct health benefit demonstrated in trials [5–7], starting ART immediately also may reduce the burden of disease by retaining in clinical care patients who would otherwise be lost to follow-up. High rates of attrition have been observed among patients who are not yet eligible for ART and who ostensibly are being monitored for disease progression, leading to missed opportunities for counseling and timely initiation of therapy [10–24]. However, the extent to which immediate ART mitigates attrition is unknown. Clinical trials, designed to minimize attrition in both arms, do not observe this phenomenon and may therefore underestimate the benefits of immediate ART. Observational studies have documented lower retention among pre-ART patients compared to patients on ART [11,12], yet these differences could simply reflect the selection of more highly-motivated patients onto ART, rather than a causal effect of ART on retention in care.

In this study, we assessed the association between immediate (versus deferred) ART eligibility and clinical retention in a large public-sector treatment program in rural South Africa. Using a quasi-experimental regression discontinuity design [25–28], we compared retention for patients presenting with CD4 counts just above and below the 350-cells/μl eligibility cutoff used during the study period. Regression discontinuity can be used when a treatment is assigned, at least in part, based on a threshold rule, such as the CD4 eligibility cutoff for HIV treatment [28]. CD4 measurements have high within-individual variation [29] due to laboratory instrument imprecision, sampling variability in blood draws, and random factors such as ambient temperature at the time of the blood draw. Due to random noise in measured CD4 counts, patients just above and below the cutoff are similar on both observed and unobserved factors, but are assigned to different exposures [28]. At the threshold, outcomes are observed in both counterfactual states of the world (eligible/not eligible), and comparisons have a causal interpretation [30]. Whereas most observational studies rely on strong assumptions about unobserved confounders, regression discontinuity can achieve balance by design, similar to a randomized controlled trial, and therefore enables causal inferences without strong assumptions [28]. This natural experiment provides a unique opportunity to assess the impact of immediate versus deferred eligibility for HIV treatment in a real-world clinical setting.

## Methods

### Ethics

Ethical approval for data collection and analysis was obtained from the University of KwaZulu-Natal Biomedical Research Ethics Committee. The research in this paper consisted of secondary analysis of preexisting de-identified data and was determined to be “not human subjects research” by the Boston University Medical Campus Institutional Review Board (H-35385, “Analysis of the HIV cascade of care in rural South Africa: A secondary data analysis”).

### Study population

The study population for this analysis consisted of all patients in the Hlabisa HIV Treatment and Care Programme (Hlabisa Cohort) [31] whose first CD4 count specimen was collected between 12 August 2011 and 31 December 2012. The Hlabisa HIV Treatment and Care Programme is a collaboration between the Africa Health Research Institute (https://www.ahri.org) and the South African Department of Health. The Hlabisa Cohort includes all patients receiving HIV care and treatment services at government facilities (17 clinics and 1 hospital) in Hlabisa sub-district, a poor, largely rural area where 1 in 3 adults is HIV-infected [32]. Patients initiating ART prior to their first CD4 count were excluded from the study.

Data on CD4 counts, viral loads, dates of ART initiation, and routine HIV clinic visits were obtained for all members of the study population. The Hlabisa HIV Treatment and Care Programme collects data on CD4 counts for patients who have not yet initiated ART, including patients who never initiate ART. Patients entered the study on the date when their first CD4 count specimen was collected for lab testing, typically the date of HIV diagnosis. Test results were transferred directly from the laboratory into the Hlabisa Cohort database. All patients were eligible to be followed for at least 12 months. Follow-up was closed on 1 January 2014.

### Outcomes

The primary outcome was 12-month retention in care, which was defined as evidence of any routine clinic visit, lab result (CD4 or viral load), or date of ART initiation within the interval 6 to <18 months after a patient’s first CD4 count, regardless of receipt of ART. By South African national guidelines, all patients would be expected to have at least 2 documented lab tests within this period (Appendix A in S1 Appendices). Although guidelines delineate semi-annual laboratory monitoring, the wide interval allows for the fact that many patients were late for appointments but still retained in care. Results were robust to narrower intervals.

The primary outcome was designed to capture all clinical contact specified by the national guidelines for pre-ART and ART care. Patients on ART had more opportunities to appear and be classified as retained due to their greater frequency of scheduled clinic visits. In sensitivity analysis, we excluded routine clinic visits and counted patients as retained at 12 months only if they had a CD4 or viral load test or initiated ART during the period 6 to <18 months after their first CD4 count.

As a secondary outcome, we assessed the presence of a CD4 or viral load test or ART start date within 6-month intervals following a patient’s first CD4 count, out to 2 years (0 to <6, 6 to <12, 12 to <18, and 18 to <24 months). Because some patients do not return for lab tests precisely every 6 months, these 6-month intervals will underestimate the proportion of patients retained. However, these analyses may inform how retention evolves over time. Analyses of retention in care out to 18 and 24 months were constrained to the subpopulations observed for that amount of time, i.e., patients presenting before 2 July 2012 (18 months follow-up) and 1 January 2013 (24 months follow-up).

### Exposures

Per South African guidelines during the study period, patients were ART eligible if their CD4 count was <350 cells/μl and/or they had a WHO stage III/IV condition [33]. After blood was drawn for a CD4 count, all patients were instructed to return to the clinic in one week to receive their result. ART-eligible patients were enrolled in several weeks of individual and group counseling and then initiated on ART. At ART initiation, patients were instructed to return for monthly clinic visits to pick up their medication and at 6 and 12 months post-initiation (and annually thereafter) for CD4 count and viral load monitoring. Patients not yet eligible for ART were referred to pre-ART care and were instructed to return every 6 months for CD4 monitoring [31].

Based on these policies, we defined two exposures. First, we defined ART eligibility as having a CD4 count below 350 cells/μl. As our second exposure, we defined ART uptake as initiation of therapy within 6 months of a patient’s first CD4 count. Not all patients who had an eligible CD4 count went on to initiate ART: some did not return for their CD4 count results, and others did not complete the counseling sessions even after eligibility was determined. Conversely, some patients with CD4 counts at or above 350 cells/μl initiated ART on account of stage III/IV HIV illness or due to provider discretion. Thus, results for our primary exposure—an ART-eligible CD4 count—have an intention-to-treat (ITT) interpretation. We defined ART uptake at 6 months because, by national guidelines, patients who did not start ART within 6 months had another CD4 count to determine eligibility.

### Study design

To determine the effect of immediate versus deferred ART eligibility on retention, we used a quasi-experimental regression discontinuity design. Regression discontinuity can be implemented when a treatment is assigned based, at least in part, on a threshold rule on a continuous assignment variable [27,28,34–36]. Though commonly used in economics [37–39], regression discontinuity has only recently made inroads in epidemiology and clinical research [26–28,40–42]. Because of random measurement error in the CD4 count laboratory assay [29], assignment to immediate versus deferred treatment is effectively random for those patients with CD4 counts near 350 cells/μl. As such, comparisons of outcomes “just above” and “just below” this threshold have a causal interpretation (see Appendix B in S1 Appendices).

Our analytic strategy, which was based on a preexisting, single, well-known clinical practice threshold, followed best practices for the conduct and reporting of regression discontinuity designs [37,38,40,43–45]. Our primary analysis tested the null hypothesis, determined a priori, that immediate (rather than deferred) ART eligibility would have no effect on retention in care. We evaluated the relationship between the value of a patient’s first CD4 count and retention in care, allowing for a discontinuity at the threshold of 350 cells/μl and different slopes on either side of the threshold. Risk differences at the threshold were estimated using local linear regression with a data-driven Imbens-Kalyanaraman bandwidth and a rectangular kernel. We assessed robustness of the results to a wide range of alternate bandwidths, following the literature (see Appendix B, pp. 3–4, in S1 Appendices) [45,46]. Results from these models are ITT effects, i.e., differences in retention for patients assigned to immediate versus deferred treatment eligibility by their CD4 count. The data-driven bandwidth selector chooses the bandwidth that minimizes the mean squared error of the difference in predictions at the threshold (i.e., the ITT effect). The goal is to identify as large a region as possible in which the conditional expectation function (relationship between CD4 count and retention) is approximately linear. The more data included, the less random error in the prediction at the threshold, but also the greater the potential for bias if the relationship is in fact nonlinear [45]. Perhaps the greatest advantage of using a data-driven bandwidth selector is that we eliminate the ability for the investigator to manipulate the results by choosing a “preferred” bandwidth. All models were estimated using a rectangular kernel, i.e., weighting observations within the window of data equally. Additionally, we estimated local logistic regression models and estimated predicted margins at the threshold. Because optimal bandwidth selectors are not currently available for logistic regression, we used the same bandwidth as for the local linear model.

We also assessed the association between immediate versus deferred eligibility and 6-month uptake of ART and used this analysis to estimate the share of patients for whom the decision to initiate ART was based on the eligibility of their CD4 count (so-called compliers) as opposed to disease stage or other factors [47]. Using an instrumental variables approach [37,48], we then estimated the effect of ART uptake on 12-month retention for compliers, using CD4 count < 350 cells/μl as an instrument for ART uptake. Under the assumption that having an eligible CD4 count affected 12-month retention only through uptake of ART, these instrumental variables estimates can be interpreted as the causal effect of ART initiation on retention among compliers (see Appendix B, pp. 5–7, in S1 Appendices). We estimated complier causal risk differences, also known as complier average causal effects (CACEs) or local average treatment effects (LATEs), using 2-stage least squares regression. We additionally estimated proportions of patients retained among patients who started ART because they were eligible (so-called treated compliers) and among patients who did not start ART because they were ineligible (so-called control compliers), and estimated complier causal relative risks as the ratio of the treated and control complier proportions [49].

The validity of the regression discontinuity design rests on the assumption that other patient characteristics that may influence retention are similar for patients with CD4 counts just above and below 350 cells/μl. To evaluate covariate balance, we assessed whether observed factors (age, sex, date of presentation, and clinic of presentation) were similar on either side of the threshold. Bias can also result if patients or providers manipulate CD4 count values in order to gain access to treatment. To assess systematic manipulation, we tested for heaping of CD4 counts on one side of the threshold [44]. All analyses were conducted using Stata/SE version 14.2. This report has been prepared according to STROBE guidelines, recommended by the Enhancing the QUAlity and Transparency Of health Research (EQUATOR) network (S1 STROBE Checklist). A de-identified analytic dataset (S1 Data) and replication code (S1 Code) are available.

## Results

### Sample characteristics

**Fig 1:**
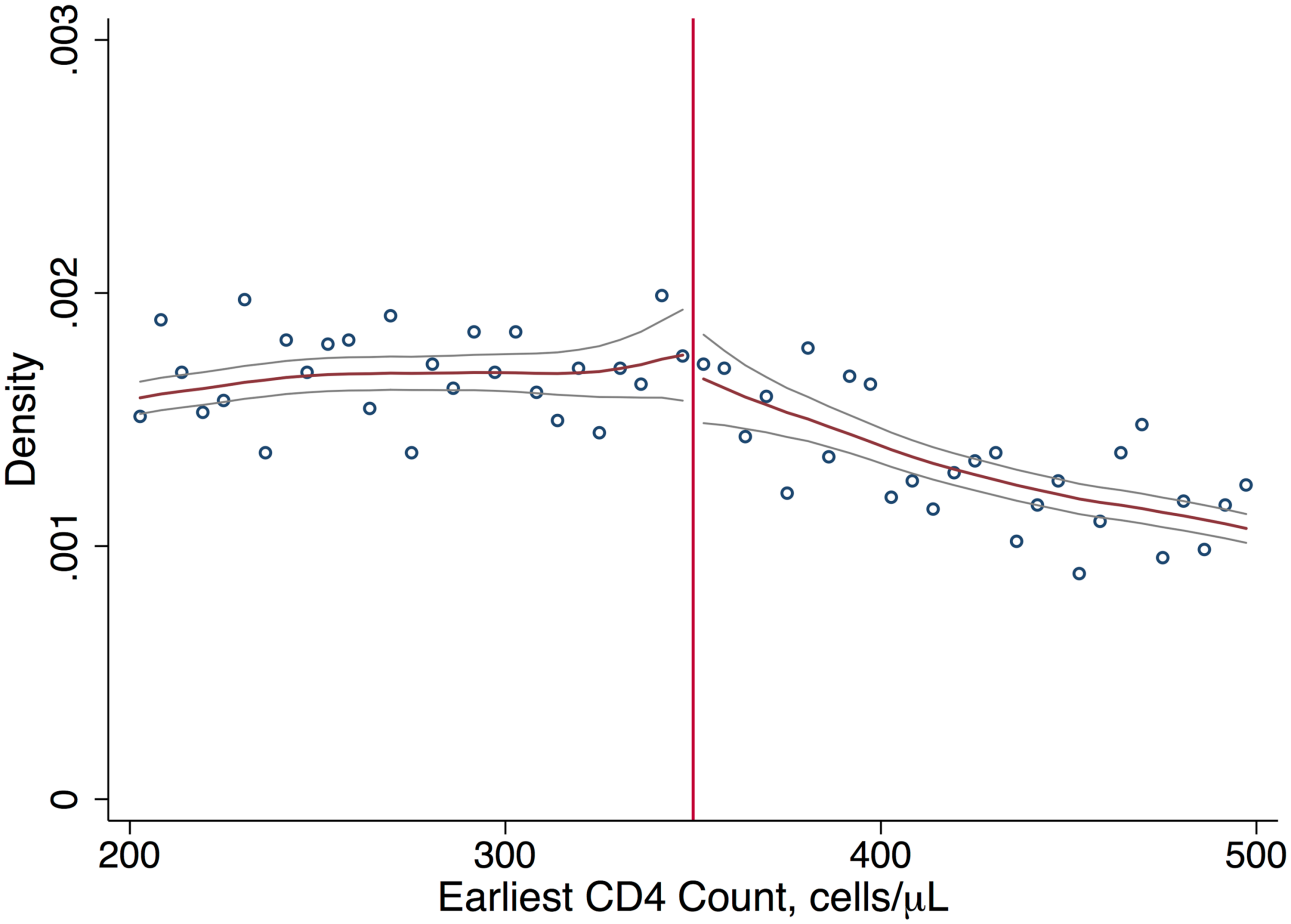
Density of first CD4 counts. Continuity in the density of first CD4 counts supports our interpretation that patients and providers did not systematically manipulate CD4 counts, e.g., to gain eligibility for ART.

A small proportion of patients (4.1%) initiated ART prior to their first CD4 count and were excluded from the study. The remaining sample included 11,306 patients who entered care with a first CD4 count between 12 August 2011 and 31 December 2012. Of these, 6,225 had CD4 counts < 350 cells/μl and 5,082 had CD4 counts ≥ 350 cells/μl. Baseline characteristics were similar just above and below the CD4 count threshold; about 70% of patients were women, and the average age was 30 years (Table 1). We found no evidence of systematic manipulation of CD4 count values around the threshold (Fig 1).

**Table 1:** Balance in baseline characteristics of patients just above and below the 350-cells/μl CD4 count threshold.

Characteristic	Predicted means for patients with:		Difference at the threshold (95% CI)	*p*-value	IK bandwidth (cells/μl)	*N*
	CD4 count just below 350 cells/μl (ART eligible)	CD4 count just above 350 cells/μl (not yet ART eligible)				
Age (years)	30.5	30.4	0.1 year (−1.2, 1.5)	0.856	124.6	4,231
Female	70.4%	72.3%	−1.9% (−6.8, 3.1)	0.453	143.4	4,825
Date of first CD4 count	13 April 2012	24 April 2012	−11.6 days (−26.9, 3.6)	0.135	153.6	5,176
Clinic A	14.1%	13.0%	1.0% (−2.8, 4.8)	0.598	142.9	4,851
Clinic B	12.2%	14.9%	−2.7% (−6.7, 1.3)	0.185	128.2	4,415
Clinic C	17.5%	13.8%	3.8% (−1.2, 8.7)	0.138	83.2	2,955

Total *N* = 11,306. Each row displays predicted means just above/below the 350-cells/μl threshold for a different baseline characteristic. The table is analogous to a balance table in a clinical trial: systematic differences between patients just above/below the cutoff would suggest that treatment assignment was not random. Predicted means were based on local linear regression models in which each baseline characteristic was regressed on first CD4 count, with different slopes on either side of the threshold and an intercept shift at the threshold. Each row is based on a separate regression model estimated using data within a window determined by the data-driven Imbens-Kalyanaraman (IK) bandwidth for that characteristic. The IK bandwidth and sample size (*N*) for each regression is reported. Although the total *N* is 11,306, the units included in each model depend on the bandwidth.

### Immediate versus deferred ART eligibility and retention in clinical care

**Fig 2:**
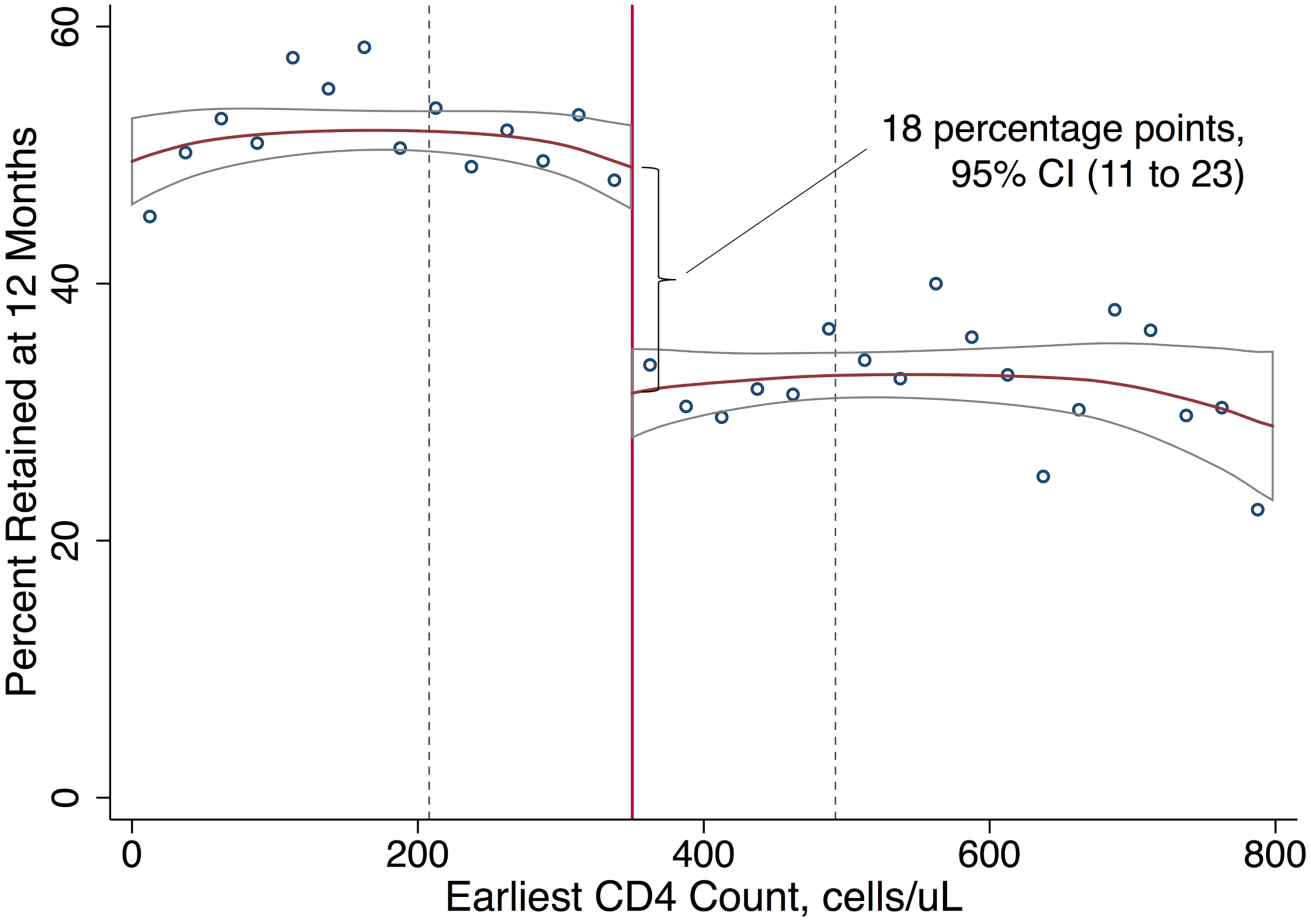
Immediate ART eligibility leads to significant gains in 12-month retention. Twelve-month retention is defined as having a CD4 count or viral load test, initiating ART, or attending a routine clinic visit within the period 6 to <18 months after first CD4 count. The sample excludes patients with <18 months of potential follow-up. Local linear regression estimated with Imbens-Kalyanaraman optimal bandwidth = 142.1 cells. The difference at the threshold was 18 percentage points (95% CI 11 to 23). The effect of interest in regression discontinuity is the difference in the local linear predictions at the threshold, i.e., in the limit, as the threshold is approached from above versus below. The bandwidth defines the region in which the relationship between first CD4 count and the outcome is assumed to be linear in our local linear regression models (Table 2).

Among patients presenting with first CD4 counts close to 350 cells/μl, immediate ART eligibility (CD4 count < 350 cells/μl) was associated with higher 12-month retention in care, with a difference of 18 percentage points (95% CI 11 to 23), relative to deferred eligibility. Fifty percent of patients with immediate eligibility were retained at 12 months compared to 32% of patients with deferred eligibility, a 56% relative increase with eligibility (Fig 2; Table 2). In sensitivity analyses defining retention in terms of lab results or ART start dates (and excluding clinic visits), estimates were somewhat attenuated, with a difference in 12-month retention of 11 percentage points (95% CI 4 to 18) (Table 2; Fig C1 in S1 Appendices). A gap in retention was observed at all 6-month intervals from first CD4 count to 24 months (Table 2; Fig C2 in S1 Appendices), suggesting that loss to follow-up among patients not yet eligible for ART occurred soon after their initial clinic visit. For all retention outcomes, results were robust to a wide range of alternate bandwidths (Tables D1–D5 in S1 Appendices). Similar results were observed using a logistic rather than linear probability model (Table D6 in S1 Appendices).

**Table 2:** Intention-to-treat effects of ART eligibility on ART initiation and retention in HIV care.

Outcome	ART initiation by 6 months	Retained at 12 months (labs, ART, clinic visits)	Retained 0–6 months (labs, ART)	Retained 6–12 months (labs, ART)	Retained 12–18 months (labs, ART)	Retained 18–24 months (labs, ART)	Retained at 12 months (labs, ART)
**Risk difference at 350-cells/μl CD4 threshold**							
Regression coefficient	25.4	17.9	17.1	8.2	4.6	9.1	11.2
95% CI	(19.7, 31.1)	(11.4, 24.3)	(11.3, 22.9)	(3.8, 12.6)	(−1.0, 10.1)	(2.4, 15.8)	(4.2, 18.1)
*p*-Value	<0.001	<0.001	<0.001	<0.001	0.108	0.007	0.002
**Predicted outcomes at 350-cells/μl CD4 threshold**							
Eligible for ART (CD4 just below 350)	43.2	49.7	47.4	28.8	21.7	19.0	41.0
Not eligible for ART (CD4 just above 350)	17.8	31.8	30.3	20.6	17.2	9.9	29.9
IK bandwidth, cells/μl	96.4	142.1	114.2	164.7	125.4	164.2	116.8
*N*	3,354	3,327	3,937	5,478	2,954	1,734	2,733

Each column reports the results of a separate linear probability regression discontinuity model, which includes an intercept, an intercept shift at the threshold, and different slopes on either side of the threshold. Results are presented on a percentage point scale (×100). The risk difference estimate shows the regression coefficient, heteroskedasticity-robust 95% CI, and *p*-value for the test that the coefficient is equal to zero. Predicted outcomes at the threshold were estimated by the constant in the regression (prediction if not eligible) and by the sum of the constant and the risk difference (prediction if eligible). Models were estimated for a window of data around the threshold equal to twice the Imbens-Kalyanaraman (IK) optimal bandwidth, which was estimated separately (and is reported separately) for each outcome. All regression coefficients, including coefficients for the slopes of the regression lines on either side of the threshold, are reported in Table D1 in S1 Appendices.

### Immediate versus deferred eligibility and ART uptake

**Fig 3:**
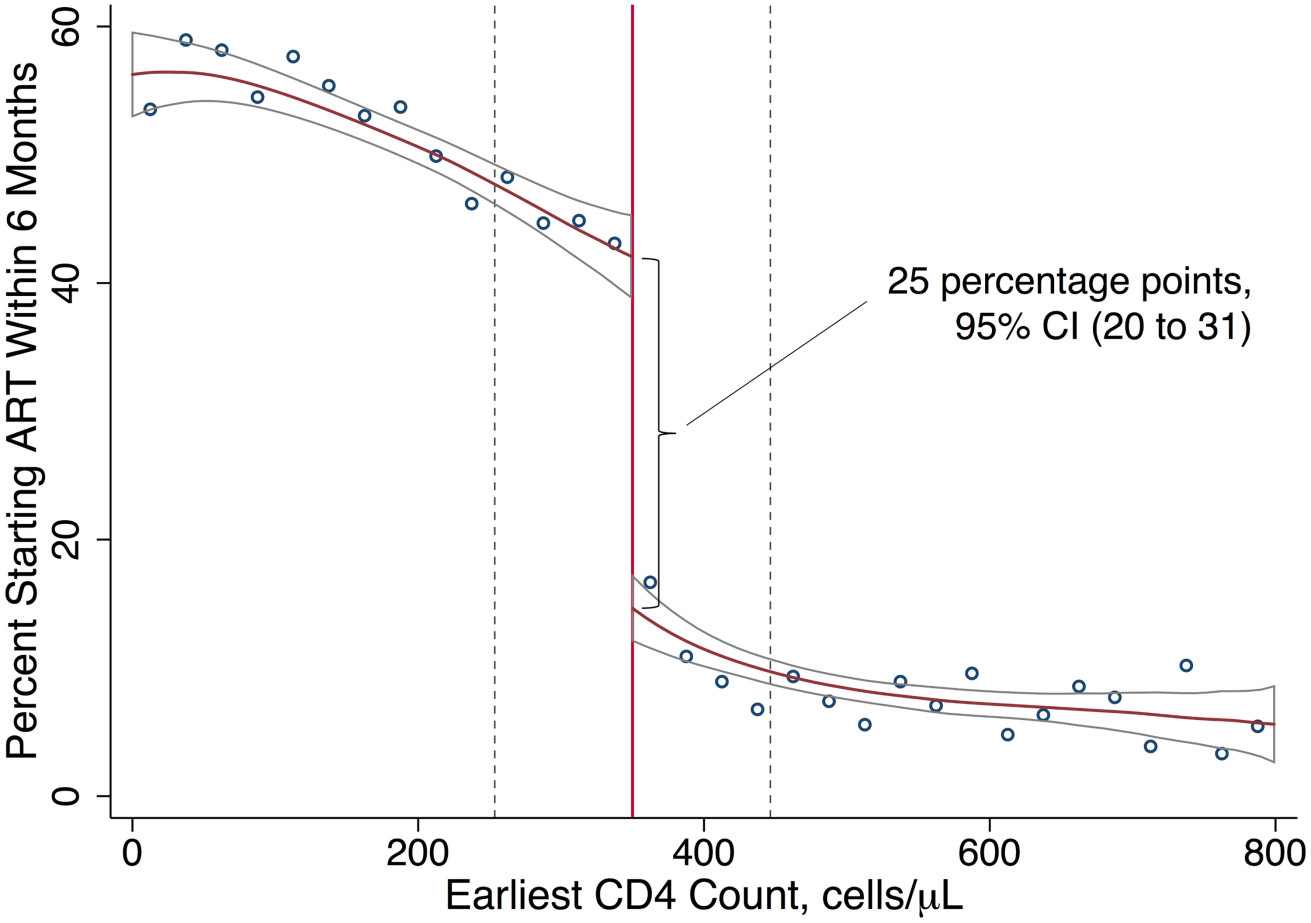
ART uptake increases with an eligible CD4 count. Local linear regression estimated with Imbens-Kalyanaraman optimal bandwidth = 96.4 cells. The difference at the threshold was 25 percentage points (95% CI 20 to 31). The effect of interest in regression discontinuity is the difference in the local linear predictions at the threshold, i.e., in the limit, as the threshold is approached from above versus below. The data-driven bandwidth refers to the region in which the relationship between first CD4 count and the outcome is assumed to be linear in our local linear regression models (Table 2).

Turning to uptake of ART, patients immediately eligible for ART were 25 percentage points (95% CI 20 to 31, *p* < 0.001) more likely to initiate ART within 6 months (Table 2) than those not yet eligible for ART, rising from 18% initiating ART among patients just above the threshold to 43% initiating ART among patients just below the threshold (Fig 3). Even among patients with an eligible CD4 count, a majority (57%) did not initiate ART within 6 months. Fig C3 in S1 Appendices shows that having an eligible CD4 count had no effect on initiation within the first 2 weeks, consistent with treatment guidelines. The gap in ART uptake apparent at 6 months persisted at 12 months. These results imply that among patients with CD4 counts close to 350 cells/μl, 18% would have initiated ART regardless of CD4 eligibility (so-called always takers), 57% would not have initiated ART regardless of CD4 eligibility (so-called never takers), and 25% of patients would have initiated ART if CD4 count < 350 cells/μl, but not if CD4 count ≥ 350 cells/μl (so-called compliers [48]) (Fig 3). Our study population was followed from the day their first CD4 count was taken, typically the day of diagnosis, regardless of whether patients returned for their results. “Never takers” therefore include patients who tested positive and never came back.

### ART uptake and retention in care among compliers

The results for ART uptake reveal that the ITT effect of CD4 eligibility on retention was substantially diluted by noncompliance—the decision to start ART was based on CD4 count for just 1 in 4 patients presenting close to the threshold. The ITT effect thus underestimates the effect of ART uptake itself on retention in care. Our instrumental variables results for compliers revealed that patients who initiated ART because they had an eligible CD4 count were 70 percentage points (95% CI 42 to 98) more likely to be retained at 12 months than patients who were not initiated because they had an ineligible CD4 count. Retention at 12 months was 91% for compliers assigned to immediate eligibility and 21% for compliers assigned to deferred eligibility. Among compliers, immediate eligibility reduced attrition by 89% (complier causal relative risk = 0.11, 95% CI 0.00–0.32) (Table 3; Table D7 in S1 Appendices).

**Table 3:** Retention in care among patients whose treatment decision was based on the value of their CD4 count.

	Point estimate	95% CI^a^
**Percent retained at 12-months**		
Patients who started ART because CD4 count < 350 cells/μl *(compliers assigned to immediate eligibility)*	91.0%	(75.8, 100.0]
Patients who did not start ART because CD4 count ≥ 350 cells/μl *(compliers assigned to deferred eligibility)*	20.8%	[0.0, 45.8)
Patients who started ART and would have started regardless of CD4 count *(always takers)*	86.9%	(77.5, 96.0)
Patients who did not start ART and would not have started regardless of CD4 count *(never takers)*	22.1%	(16.5, 28.6)
**Absolute and relative effect estimates**		
CACE^b^—percentage point difference in retention	70.2	(40.9, 100.0]
CCRR^c^—relative risk of attrition	0.11	[0.00, 0.32)

*N* = 2,366. Estimates calculated based on local linear regression models with a bandwidth of 100 cells/μl. Details on estimation of percent retained among subgroups are provided in Appendix D in S1 Appendices. Percent retained among compliers assigned to deferred eligibility was obtained by subtracting CACE from the percent retained among compliers assigned to immediate eligibility.

^a^All 95% CIs were obtained using the percentile bootstrap (501 resamples). Square brackets are used where the bootstrap CI exceeded logical bounds, e.g., probability less than zero. For CACE, the percentile bootstrap CI was similar to the standard asymptotic 95% CI using heteroskedasticity-robust standard errors: (42.3, 98.1). Both CACE and CCRR were significantly different from the null hypothesis of no effect, *p <* 0.001.

^b^CACE is interpretable as a risk difference and is also known as the local average treatment effect (LATE). CACE was estimated by 2-stage least squares regression.

^c^CCRR is presented in terms of the risk of attrition (1 − retention): among compliers, immediate eligibility reduced attrition by 89%.

CACE, complier causal relative risk; CCRR, complier causal relative risk.

## Discussion

Understanding the extent to which immediate ART initiation mitigates loss of health and life due to failure to remain in care is important for countries and funding agencies considering WHO recommendations to start patients immediately on therapy regardless of CD4 count [8,9]. Although lower retention has been observed in pre-ART patients compared to ART patients in a wide range of settings, it was hitherto unknown whether this reflected a causal relationship or selection of highly motivated patients onto ART [11,12]. If starting ART causally increased retention in care, then the real-world benefits of immediate ART would be underestimated in clinical trials that actively retain patients not yet eligible for therapy [5–7].

Using a quasi-experimental regression discontinuity design, we found that having an ART-eligible CD4 count at diagnosis significantly improved retention in care for HIV patients in rural South Africa—by 18 percentage points in the ITT analysis. The ITT effect was diluted by the fact that the decision to start ART was based on CD4 count for only a minority of patients (just 1 in 4) presenting for care. (Other patients started ART due to disease stage, while others did not start ART in spite of having an eligible CD4 count.) Among patients whose treatment decision was based on their CD4 count, immediate ART eligibility increased 12-month retention by 70 percentage points relative to deferred eligibility, from 21% to 91% retained.

The retention advantage for ART-eligible patients is perhaps surprising. Clinical guidelines specified that patients who did not start ART should return for CD4 monitoring every 6 months to reassess eligibility. One might expect that patients who are motivated to start lifelong ART would tolerate a 6-month delay without exiting care. Further, because the lifetime benefits of ART are greater the earlier a patient starts therapy, there is a strong rationale to stay in pre-ART care in order to initiate as soon as possible. Conceivably, the retention advantage could even favor pre-ART patients as some patients who initiate ART subsequently exit care after experiencing drug side effects and the inconvenience of daily therapy. With respect to the burdens imposed on patients, it might be easier to retain patients on a holding regimen of semi-annual pre-ART appointments than to retain patients on an intensive daily drug therapy. Patients who do not start ART also might be more likely to get sick and need care 12 months later, leading to higher retention in this group.

Contrary to these speculations, immediate eligibility for ART sharply increased retention in care. There are several plausible explanations for the observed results. First, starting ART may shift patients’ cognitive reference point for future care-seeking decisions [50]. Patients who have started ART may perceive large costs to defaulting therapy, compared to the more modest costs of delaying initiation among those who have not yet started therapy. These perceptions may be reinforced by clinical guidance to patients that starting ART involves a commitment to take treatment for life [31]. Second, taking daily therapy and returning to the clinic for monthly prescription refills may facilitate habit formation [51], increasing long-term retention in care. Third, auxiliary interventions targeted to ART patients including adherence counseling, support groups, appointment reminders, and outreach to patients who miss appointments may lead to differential retention between patients on ART and those not yet on ART [52,53]. Fourth, in the context of still-rampant HIV stigma, fear of HIV status disclosure may be a significant barrier to care-seeking among patients not yet on ART [54,55]. Many clinics strongly encourage patients to disclose their HIV status to close family and friends at the time of ART initiation [31]. For ART patients, HIV status disclosure may represent an up-front investment that reduces the costs of future clinic visits. In addition to the behavioral mechanisms above, a fifth possibility is that patient health and quality of life improve due to the antiretroviral drugs themselves [56] and that experiencing these benefits encourages patients to remain in care.

In addition to the benefits of immediate ART eligibility, it is also possible that patients who are told that they are not yet eligible for ART may be inadvertently discouraged from seeking care in the future. The message of deferred eligibility may falsely signal to patients that they do not need or would not benefit from ART. They may also experience anger, hopelessness, or demoralization if they wish to start therapy but are not allowed to, and these experiences may color their attitudes towards the health system and future care-seeking.

Our results highlight the challenges in retaining in care those patients who test positive but are not yet eligible for ART in a resource-limited setting [57]. And yet, the high rates of retention among patients initiated on ART because they were eligible suggest that, in fact, we already have effective techniques to improve retention among patients who have not yet started ART. Further research is needed to identify what specific aspects of initiating ART lead to improved retention in care. These findings may be important for retaining patients who do not wish to start ART on the day of diagnosis, as is now called for in 2017 WHO guidelines [58].

### Causal interpretation of the estimates

Our results were obtained using a regression discontinuity design, a quasi-experimental study design that enables causal inference without the strong assumptions required in most observational studies [26–28,35,40,43,59–61]. So long as values of CD4 measurements are not systematically manipulated by patients or providers, random variability in measured CD4 counts guarantees that patients will be similar (in expectation) in a small range on either side of the 350-cells/μl eligibility threshold [30,35]. We obtained CD4 counts directly from laboratory records (rather than clinical charts) and found no evidence of manipulation, which, if systematic, would lead to heaping in the density of CD4 counts on one side of the threshold [44]. Additionally, there were no systematic differences in observed baseline covariates between patients just above versus below the cutoff. Although we cannot test the assumption that unobserved factors are balanced at the cutoff, our knowledge of the assignment mechanism, the absence of systematic manipulation, and balance on observed characteristics all point to a data-generating process in which quasi-random variation guarantees balance on all factors, similar to a randomized trial.

As with all regression discontinuity designs, causal effects are theoretically identified at the threshold (i.e., in the limit, as the 350-cells/μl cutoff is approached from above and below). In finite samples, however, these causal effects must be estimated using data further from the threshold. We followed current best practice in using local linear regression to estimate the empirical relationship between measured CD4 count and the probability of retention, allowing for an intercept shift at the threshold and different slopes on either side of the threshold [46,62]. The intercept shift at the threshold—i.e., the difference in regression predictions just above and just below 350 cells/μl—estimates the causal effect at the threshold. When using a local linear model to approximate a potentially nonlinear relationship, a key choice is the bandwidth governing the window of data used in the analysis. While a larger bandwidth will increase the precision of the estimates, this may come at the cost of some bias. We used the data-driven Imbens-Kalyanaraman optimal bandwidth selector, which minimizes the mean squared error (variance plus squared bias) of the difference in predictions at the threshold [45].

In many classical applications of the regression discontinuity design, the assignment variable is associated with the outcome and with the size of the treatment effect. Interestingly, retention was not substantially correlated with first CD4 count in our sample, and slopes were similar on either side of the threshold, evidence that treatment effects may be constant, at least within a range around the 350-cells/μl cutoff. Because we did not know this ex ante, we nevertheless present results from models allowing for different slopes and constrain inferences to the area around the cutoff. By choosing a local effect estimand, our estimates do not rely on extrapolation into unobserved regions nor on assumptions about the functional form of the relationship between CD4 count and retention across the full range of the data [35].

In addition to estimating the ITT effect of ART eligibility on retention, we also estimated the effect of ART initiation itself on retention in care using the eligibility threshold as an instrumental variable. These estimates are interpretable as the effect of starting ART for so-called compliers, i.e., those patients for whom the decision to start (or not to start) ART was based on the value of their CD4 count vis-à-vis the 350-cells/μl threshold. These instrumental variables estimates have a causal interpretation under two additional assumptions. The first assumption, known as the monotonicity or “no defiers” assumption, is that having an eligible CD4 count only increases the chances that a person will start ART. Monotonicity would be violated if there are patients who would start ART if they were ineligible (CD4 ≥ 350 cells/μl) but would not start ART in a counterfactual world in which they had an ART-eligible CD4 count. It is difficult to conceive of such cases, and this assumption is likely met in our study. The second assumption, known as excludability, is that eligibility differences at the 350-cells/μl threshold affect retention only through ART uptake. This untestable assumption could be violated if eligibility led to other differences in care apart from ART initiation (e.g., screening for other conditions or pre-ART counseling) that led to increases in later engagement with care. Monotonicity and excludability assumptions are not required for a causal interpretation of the ITT result.

A key strength of regression discontinuity designs (vis-à-vis clinical trials) is the ability to assess the causal effects of interventions implemented in real-world settings and in population-representative samples [63]. We studied the complete patient population accessing public-sector HIV care and treatment in one of the poorest and highest HIV-prevalence sub-districts in South Africa. Although our analysis was limited to one sub-district of one country, potentially limiting generalizability, the model of service delivery—decentralized, nurse-led, clinic-based—is common in other HIV-endemic areas of sub-Saharan Africa. Additionally, by including the complete patient population, we avoided the sample selection bias that can result from opt-in participation in clinical trials [64].

Our retrospective analysis of a quasi-experiment avoided many of the potential pitfalls that can lead to bias when investigators knowingly or unknowingly affect outcomes. First, the CD4 count threshold we investigate was set by policy-makers in advance of the study and could not be manipulated by the investigators. Second, patients, providers, and investigators were all blinded to the CD4 count measurement (and hence eligibility status) of the patients at the time when blood was drawn for the patients’ first CD4 count. Because we obtained the CD4 results for all blood samples directly from the National Health Laboratory Service, it would have been very difficult for eligibility assignment to be manipulated. Third, the data that we analyzed were collected as part of routine laboratory and clinical monitoring of patients in the Hlabisa HIV Treatment and Care Programme, and thus were not vulnerable to Hawthorne effects or other investigator biases in collection. Fourth, our analytic approach—local linear regression using a data-driven optimal bandwidth—is a theory-driven and widely used best practice in the conduct of regression discontinuity studies [37,38,40,43–45] and was decided on *a priori*. By using a data-driven bandwidth selector, we eliminated an opportunity for the investigator to manipulate the results by choosing a “preferred” bandwidth. By choosing local linear regression *a priori*, we avoided investigator discretion in the choice of functional form. Finally, following guidelines for the conduct of regression discontinuity studies, we assessed the data for evidence of manipulation of the assignment variable and conducted tests for balance at the threshold on all baseline characteristics observed and available in the dataset. We found no evidence to suggest that patients were dissimilar just above and below the treatment threshold.

### Limitations

Our study has some limitations. One limitation is that, as discussed above, our local linear regression results may be biased if the relationship between earliest CD4 count and retention in care is nonlinear near the threshold. Nonlinearities were taken into consideration when choosing the bandwidth (the window of data) for the model. Additionally, visual inspection of our figures suggests that in fact the relationships were approximately linear and that any bias resulting from using a linear model would be very small relative to the size of the effect estimates. Our results were also robust to using smaller bandwidths.

A second limitation involves the generalizability of our estimates to patients presenting with different CD4 counts and to different patient populations. As with any regression discontinuity design [26,28,36,41], our results are interpretable as causal effects for patients presenting with CD4 counts close to the 350-cells/μl eligibility threshold. If treatment effects differed across CD4 counts, then our results would not be directly informative of effects at higher (or lower) CD4 counts. Although this is a limitation, it is likely that our estimates are broadly generalizable to other points in the CD4 count distribution. The probability of retention changes little with the value of the patient’s first CD4 count, except at the threshold, and prior analysis showed similar effects at the 200-cells/μl eligibility threshold used prior to August 2011 [42]. Our analysis was also limited to one sub-district of one country, and it is unknown whether the results generalize to other settings.

A third limitation regards the difficulty of measuring retention. There are competing definitions in the literature [65]. In our primary specification, we classified patients as retained if they had any routine contact with the clinic within 6 to <18 months after first CD4 count, including visits to pick up ART medication, laboratory tests (CD4 or viral load), or an ART initiation date, all of which are specified as elements of routine pre-ART or ART care. As a robustness check, we defined an alternate measure of retention based only on laboratory tests and dates of ART initiation. We note that by excluding routine visits, this measure underestimates retention among ART patients and should be interpreted as a lower bound.

A fourth limitation of our analysis is that we were unable to assess longer-term health outcomes that may result from poor retention. In previous analysis, we found large survival benefits of immediate ART eligibility for patients presenting with CD4 counts near the former eligibility threshold of 200 cells/μl [28,42]. However, it is unknown whether these survival benefits extend to patients presenting at higher CD4 counts. We observed significant gaps in retention at 18–24 months. Patients whose CD4 counts are not actively monitored for treatment eligibility may initiate long after their CD4 count falls below the eligibility threshold, or they may not initiate at all. Extended treatment delays have consequences not only for patients themselves but also for population health, with increased potential for onward transmission [66]. Further research will be needed to determine the real-world impacts of deferred ART at higher CD4 counts on long-term engagement with care, health, survival, and onward transmission.

### Implications and next steps

**Fig 4:**
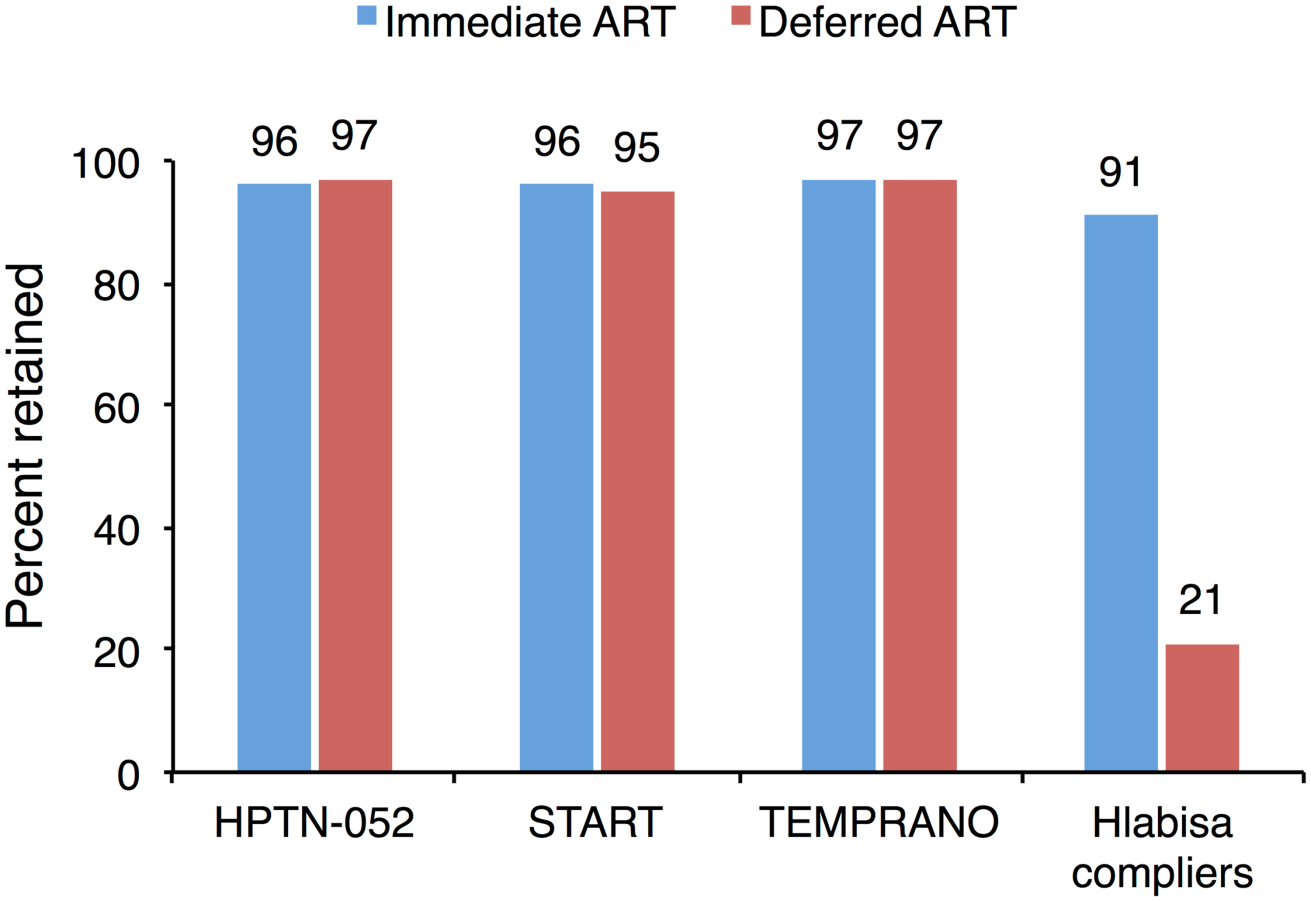
The effect of immediate ART on retention is not observed in clinical trials. Retention is reported at 2.1 years for the HPTN-052 trial [5], 3 years for the INSIGHT START trial [7], 3.5 years for the TEMPRANO trial [6], and 12 months for Hlabisa. Estimates for Hlabisa are for compliers—those patients whose treatment decision was determined by the value of their CD4 count.

International treatment guidelines are informed (largely) by clinical trials [8,47,58,67], which typically differ from clinical care in the “real world” in important dimensions. One of these dimensions is retention in care. Clinical trials usually seek to retain patients in all treatment arms through systematic monitoring and outreach efforts. For example, in the TEMPRANO trial, 30-month retention was 97% in both arms [6]. The parity of retention across arms in these trials stands in stark contrast to the very large gap—91% versus 21%—we observed in a non-trial setting (Fig 4). Efforts to minimize attrition improve the validity of inferences on clinical endpoints; however, trials cannot then observe the effect of the intervention on retention in care, nor any downstream health impacts that are mediated through retention [5–7,68,69]. In this and other applications, the gap between clinical efficacy (as demonstrated in clinical trials) and real-world effectiveness may turn on the nature of the relationship between the intervention and retention of patients in care—a question of patient behavior, not biology.

Prior observational studies have documented higher retention among ART patients than among pre-ART patients [11,12,23,24]; however, this association is difficult to interpret due to potential selection bias. If highly motivated patients were more likely to initiate ART—leaving a residual of less motivated patients in pre-ART care—then a policy expanding ART eligibility may simply shift patients with low motivation from pre-ART to ART, leading to low retention and poor outcomes for newly eligible patients on treatment. On the other hand, if initiating patients on ART causally increases retention in care, then immediate ART eligibility would improve retention, leading to even larger benefits than observed in clinical trials. Our results provide evidence, to our knowledge for the first time, to distinguish between these two competing hypotheses. We demonstrate a large difference in retention between pre-ART and ART patients, causally attributable to starting ART itself.

The gap in retention observed in this study would be eliminated if patients were eligible for ART regardless of CD4 count, as under test-and-treat scenarios now being implemented in many countries. We caution that some patients may have little interest in initiating therapy even if eligible [59]. Our results are not informative about the impact of immediate therapy for this group, and increasing demand for ART in such patients may be a challenge. Nor do our estimates generalize to the smaller group of patients who start ART for other reasons (e.g., disease stage) regardless of CD4 count. But for those patients currently barred from initiating due to an ineligible CD4 count, we show that a guideline change allowing immediate initiation could dramatically increase retention in care. Early WHO guidelines for HIV were designed to prioritize the sickest for ART, and there has been concern that expanding eligibility would inappropriately target resources to patients with little incentive to remain on therapy [70]. Our results suggest that expanding eligibility would target patients who are both high need (only 21% would be retained if not eligible) and high performing (91% would be retained if eligible). Countries such as South Africa [9], which have now removed CD4 thresholds, can be encouraged that such a policy will be an efficient step towards expanding HIV treatment coverage.

### Conclusion

Clinical trials have demonstrated the biological efficacy of early ART [5–7]. Effects on patient retention, however, cannot be observed in trials that minimize attrition by design. Our study demonstrates, to our knowledge for the first time, the retention effects of early ART: denying ART eligibility to patients who would be willing to start therapy leads to very large losses from HIV care, losses that would be avoided with immediate ART. Our results thus indicate that the real-world benefits of extending ART eligibility to all patients, regardless of CD4 count, may be larger than previously thought.

## Supporting information

S1 Appendices Supporting information on the study methods, supplementary figures, and supplementary tables. Appendices A and B: supporting information on study methods. Appendix C: supplementary figures. Appendix D: supplementary tables.
(DOCX)

S1 Code Replication code (Stata/SE version 14.2.do file). (DO)

S1 Data Description of replication dataset and how to access it. (DOCX)

S1 STROBE Checklist (DOC)

## Abbreviations

ART: antiretroviral therapy
CACE: complier average causal effect
ITT: intention-to-treat
