# Supplementary material for: Treatment eligibility and retention in clinical HIV care: A regression discontinuity study in South Africa
Source: PLoS Med. 2017 Nov 28;14(11):e1002463. doi: 10.1371/journal.pmed.1002463 (PMC5705070; doi:10.1371/journal.pmed.1002463)
Supplement: S1 Appendices — Appendices A and B: supporting information on study methods. Appendix C: supplementary figures. Appendix D: supplementary tables. (DOCX) [file pmed.1002463.s001.docx]

**APPENDICES**

**Treatment eligibility and retention in clinical HIV care: regression-discontinuity evidence from South Africa**

Jacob Bor, ScD, Matt Fox, PhD, Sydney Rosen, MPA, Atheendar Venkataramani, MD/PhD, Frank Tanser, PhD, Deenan Pillay, PhD, Till Bärnighausen, MD/PhD

Correspondence to: J Bor [jbor@bu.edu](mailto:jbor@bu.edu).

**CONTENTS**

- Appendix A: Description of lab-test scenarios p2
- Appendix B: Description of Methods p3-10
- Appendix C: Supplementary Figures p11-13
- Appendix D: Supplementary Tables p14-20

**Appendix A: Description of lab-test scenarios**

The following scenarios describe the timing of lab tests that would be conducted according to guidelines for patients with different eligibility statuses. Under all scenarios a patient would be expected to have at least two laboratory tests in the interval 6-18 months after first CD4 count. Time from eligibility to initiation is assumed to be two months in these scenarios.

*Scenario 1: baseline eligible*

- initiates at 2 months
- first VL/CD4 at 8 months
- second VL/CD4 at 14 months

*Scenario 2: baseline ineligible, but eligible at next CD4 count*

- first CD4 at 6 months, eligible
- initiates at 8 months
- first VL/CD4 at 14 months

*Scenario 3: baseline ineligible, ineligible at next CD4, but eligible at next CD4*

- first CD4 at 6 months
- second CD4 at 12 months, eligible
- initiates at 14 months

*Scenario 4: never eligible*

- first CD4 at 6 months
- second CD4 at 12 months
- third CD4 at 18 months

**Appendix B: Description of Methods**

**Regression Discontinuity Design**

Our study design exploits the fact that patients with CD4 counts just above/below the 350-cell/μL threshold are similar on all observed and unobserved factors, but assigned to different exposures (eligible vs. not eligible for ART). How does this natural experiment arise? Measured CD4 counts are notoriously imprecise,[1] reflecting a combination of measurement error, sampling variability in blood draws, and biological fluctuations. For patients with true CD4 counts arbitrarily close to 350 cells, random noise in CD4 count measurements randomly assigns patients to be on one side of the eligibility threshold or the other.[2,3] Random noise guarantees continuity in all potential confounders across CD4-count measurements. Thus, in the limit, patients on either side of the threshold are identical (in expectation) and regression models can be used to predict outcomes just above and below the threshold.[4] Whereas other observational study designs must rely on strong assumptions about unobserved confounders, regression discontinuity can achieve balance by design, similar to an RCT.[5]

Following the literature, differences in outcomes at the threshold were estimated using local linear regression.[6] A preliminary bandwidth was chosen using the Imbens-Kalyanaraman (IK) optimal bandwidth, which minimizes the mean-squared error of the difference at the threshold.[7] We used a rectangular kernel for our local linear models, which is identical to linear regression on a window of data around the threshold of width twice the bandwidth. Robustness was assessed relative to a wide array of bandwidths, from +/-50 to +/-200 CD4 cells. We estimate regression models of the form:

EQUATION 1

$$E[Y_{i}|Z_{i}]=\beta_{0}+\beta_{1}(Z_{i}-350)+\beta_{2}1[Z_{i}<350]+\beta_{3}(Z_{i}-350)*1[Z_{i}<350]$$

where Z_i_ is the value of a patient’s first CD4 count, 1[Z_i_<350] is an indicator variable for whether the patient’s first CD4 count was below the 350 cell/uL threshold, and Y_i_ is the outcome. $\beta_{2}=E\left[ Y_{i} | Z_{i}\uparrow350 \right]-E\left[ Y_{i} | Z_{i}\downarrow350 \right]$ is the intent-to-treat effect of having an eligible CD4 count for patients close to the threshold.

**Support for Regression Discontinuity Assumptions**

Causal inference in RDD depends on the similarity of patients on either side of the threshold. This could be jeopardized if CD4 count values were systematically manipulated, e.g. in order to gain access to treatment. Such manipulation would result in heaping of CD4 counts on one side of the threshold and a discontinuity in the density of CD4 counts at the threshold. We found no evidence of systematic manipulation of CD4 count values around the threshold (Figure 1) and could not reject the hypothesis that the density was continuous at the threshold (P = 0.542).[8]

Evidence for the similarity of patients on either side of the threshold can also be obtained from observable baseline characteristics. Patients on either side of the threshold were similar on age, sex, and date of presentation (Table 1). (Limited covariate information is available on patients at the time of their first CD4 count.) As in an RCT, similarity in observed covariates does not guarantee that there is similarity in unobserved factors. However, balance on observables provides support for our interpretation of the data generating process, which, if true, would guarantee balance on both observed and unobserved factors, in expectation.

**Intent-to-treat vs. complier causal effects**

The effect of CD4 count eligibility is an intent-to-treat effect. As in a randomized trial, the intent-to-treat effect underestimates the effect of actually starting ART. We additionally estimated the causal effect of ART uptake (initiation within six months) on retention among compliers, i.e. patients whose CD4 count determined uptake of ART.

Not all patients with an eligible CD4 count start therapy (so-called “never-takers”[9]). Conversely, some patients initiate ART regardless of CD4 count (so-called “always-takers”[9]). Only for so-called “compliers” does having an eligible CD4 count determine whether a patient initiates therapy or not.[9] We define “treated compliers” as those patients who were induced to initiate ART because they had an eligible CD4 count but would not have initiated if not eligible and “untreated compliers” as those patients who were prevented from initiating ART because they had an ineligible CD4 count but who would have initiated if eligible. Whether a patient is an always-taker, never-taker, or complier can be viewed as a latent (unobserved) characteristic that determines how the patient will respond to having an eligible CD4 count. Under the data generating process that gives rise to a valid RDD, the distribution of patients across these groups will be the same just above/below the threshold.

Complier average causal effects (also known as local average treatment effects) are computed by using eligibility as an instrumental variable for ART uptake, and are identified under two important but plausible assumptions: (1) *excludability*, CD4 count eligibility only affects retention through ART uptake; (2) *monotonicity*, no patient who would initiate if ineligible would refuse initiation if eligible (and vice-versa).[9] In the regression discontinuity literature, the use of the threshold rule as an instrument is known as “fuzzy RD” because not all patients are affected by the threshold rule.

The monotonicity assumption is very likely to be met in our setting. Violations of monotonicity would occur if there were patients who “defy” their treatment assignment: patients who would not initiate ART if eligible but who would initiate ART if not eligible. It is difficult to see how this would arise. The excludability assumption is a stronger assumption in this setting. Excludability requires that having an eligible CD4 count affected retention at 6-18 months only through differences in ART uptake at six months. This assumption could be violated if, e.g., patients who were eligible and did not start ART were more likely to be retained than patients who were not eligible and did not start ART. Such a violation is conceivable, e.g., if patients who were eligible but did not start received some additional counseling that had a direct effect on retention. However, it is likely that any such effect is small. If eligible patients who did not initiate were more likely to be retained in care, then this should translate into increased ART uptake after six months among those who were baseline eligible. Very little increase is observed (Figure C3).

**Estimating Complier Causal Effects**

We estimated the complier average causal effect (CACE) by two-stage least squares:[10]

EQUATION 2

$$CACE_{RD}=\left[ Y_{i}\left( 1 \right)-Y_{i}\left( 0 \right) | complier,Z_{i}=c \right]$$

$$=\frac{\text{Intent-to-treat effect} |Z_{i}=c}{\Pr\left( complier \right)|Z_{i}=c}$$

$$=\frac{E\left[ Y_{i} | Z_{i}\uparrow c \right]-E\left[ Y_{i} | Z_{i}\downarrow c \right]}{E\left[ T_{i} | Z_{i}\uparrow c \right]-E[T_{i}|Z_{i}\downarrow c]}$$

where Y is the outcome (e.g. 12-month retention), Z is the assignment variable (e.g., first CD4 count), c is the threshold (e.g. 350 cells), and T is an indicator for treatment uptake (e.g. ART initiation within six months).

**Estimation of Treated and Untreated Complier Means**

For a binary outcome, CACE is interpretable as a complier risk difference. However, a risk difference can be difficult to interpret on its own: e.g., a 10 percentage point increase from a baseline of 90% is very different from a 10 percentage point increase off a baseline of 40%. In addition to CACE, we also estimated proportions retained in care for “treated compliers” and “untreated compliers” separately (CACE is the difference between these proportions).[11,12] Doing so requires estimating a separate “conditional on treated” regression discontinuity model, i.e. estimating the “effect” of eligibility for patients who would go on to initiate ART in six months. Details are provided below. The complier causal relative risk (CCRR) can be constructed as the ratio of treated and untreated complier proportions. For all analyses of complier causal effects we used local linear regression with a rectangular kernel and a bandwidth of 100 cells/μL.

In the setting of a binary instrument, a binary treatment, and no covariates, distributions of outcomes are separately identified for treated and untreated compliers, as follows.[11,12] For treated compliers, mean outcomes are:

EQUATION 3

$$E\left[ Y_{i}\left( 1 \right) | complier \right]=\frac{E\left[ Y_{i} | T=1,complier or AT \right]*\Pr\left( complier or AT \right)-E\left[ Y_{i} | AT \right]*\Pr\left( AT \right)}{\Pr\left( complier \right)}$$

$$=\frac{E\left[ Y_{i} | T_{i}=1,Z_{i}\uparrow c \right]*\Pr\left( T_{i}=1 | Z_{i}\uparrow c \right)-E\left[ Y_{i} | T_{i}=1,Z_{i}\downarrow c \right]*Pr(T_{i}=1|Z_{i}\downarrow c)}{\Pr\left( T_{i}=1 | Z_{i}\uparrow c \right)- Pr(T_{i}=1|Z_{i}\downarrow c)}$$

where AT denotes “always-taker”. The terms $E\left[ Y_{i} | T_{i}=1,Z_{i}\uparrow c \right]$ and $E\left[ Y_{i} | T_{i}=1,Z_{i}\downarrow c \right]$ can be estimated in a conditional-on-treated regression discontinuity model regressing the outcome on the assignment variable (i.e., limiting the sample to the treated); the other terms on the right hand side are estimated in a regression discontinuity model in which the treatment indicator (ART uptake) is regressed on the assignment variable. The untreated complier mean can be similarly estimated using information from a conditional-on-untreated regression model.

EQUATION 4

$$E\left[ Y_{i}\left( 0 \right) | complier \right]=\frac{E\left[ Y_{i} | T_{i}=0,complier or NT \right]*\Pr\left( complier or NT \right)-E\left[ Y_{i} | NT \right]*\Pr\left( NT \right)}{\Pr\left( complier \right)}$$

$$=\frac{E\left[ Y_{i} | T_{i}=0,Z_{i}\downarrow c \right]*\Pr\left( T_{i}=0 | Z_{i}\downarrow c \right)-E\left[ Y_{i} | T_{i}=0,Z_{i}\uparrow c \right]*\Pr\left( T_{i}=0 | Z_{i}\uparrow c \right)}{\Pr\left( T_{i}=0 | Z_{i}\downarrow c \right)-\Pr\left( T_{i}=0 | Z_{i}\uparrow c \right)}$$

$$=E\left[ Y_{i}(1) | complier \right]-CACE_{RDD}$$

where NT denotes “never-taker”.

The difference between these estimates is the complier average causal effect. Therefore, treated and untreated complier means can be (i) estimated using separate conditional-on-treated and conditional-on-untreated regressions; (ii) estimated in a conditional-on-treated regression and then subtracting off the complier average causal effect (risk difference) estimated in the original full sample model; or (iii) estimated in a condition-on-untreated regression model and then adding the complier average causal effect. Because the conditional-on-treated and conditional-on-untreated models are estimated for different sub-samples, the estimates of treated and control complier means may differ slightly depending on which approach is used (i), (ii), or (iii). To maintain consistency across estimates presented, we present (ii) as our main result, subtracting off the CACE estimate from Equation 2 from the treated complier mean estimated in Equation 3. We also present results for all the underlying regressions in Table D7, from which (i) and (iii) can be computed. Results were similar across all three methods.

Having obtained treated and control complier means (proportions retained in care), the ratio of these quantities yields the complier causal relative risk, CCRR = $E\left[ Y_{i}(1) | complier \right]/E\left[ Y_{i}(0) | complier \right]$. Relative risks are commonly presented for “bad” outcomes (attrition, as opposed to retention in care). Therefore, for ease of interpretation, we present the CCRR of *attrition*, which is simply CCRR_attrition_ = $\frac{\left( 1-E\left[ Y_{i}\left( 1 \right) | complier \right] \right)}{\left( 1-E\left[ Y_{i}\left( 0 \right) | complier \right] \right)}$. CCRR_attrition_ is interpretable as the relative reduction in attrition due to early ART among patients who started ART because they had an eligible CD4 count. 95% confidence intervals were constructed for CCRR and treated and control complier means using the percentile bootstrap, with 501 resamples.

**REFERENCES**

1. Hughes MD, Stein DS, Gundacker HM, Valentine FT, Phair JP, Volberding PA. Within-subject Variation in CD4 lymphocyte count in asymptomatic human immunodeficiency virus infection: Implications for patient monitoring. J Infect Dis. 1994;169: 28–36.

2. Bor J, Moscoe E, Bärnighausen T. Three approaches to causal inference in regression discontinuity designs. Epidemiology. 2015;26: e28-30. doi:10.1097/EDE.0000000000000231

3. Bor J, Moscoe E, Bärnighausen T. The “Natural Experiment” in Regression Discontinuity Designs: Randomization without Controlled Trials (Rapid Response). BMJ. 2014;349: g5293. Available: http://www.bmj.com/content/349/bmj.g5293/rr/764146

4. Lee DS. Randomized experiments from non-random selection in U.S. House elections. J Econom. 2008;142: 675–697. doi:10.1016/j.jeconom.2007.05.004

5. Bor J, Moscoe E, Mutevedzi P, Newell M-L, Bärnighausen T. Regression discontinuity designs in epidemiology: Causal inference without randomized trials. Epidemiology. 2014;25: 729–737. doi:10.1097/EDE.0000000000000138

6. Hahn J, Todd P, Van der Klaauw W. Identification and estimation of treatment effects with a regression-discontinuity design. Econometrica. 2001;69: 201–209.

7. Imbens GW, Kalyanaraman K. Optimal bandwidth choice for the regression discontinuity estimator. Rev Econ Stud. 2011;79: 933–959. doi:10.1093/restud/rdr043

8. McCrary J. Manipulation of the running variable in the regression discontinuity design: A density test. J Econom. 2008;142: 698–714. doi:10.1016/j.jeconom.2007.05.005

9. Imbens GW, Angrist JD. Identification and estimation of local average treatment effects. Econometrica. 1994;62: 467–475.

10. Imbens GW, Lemieux T. Regression discontinuity designs: A guide to practice. J Econom. 2008;142: 615–635. doi:10.1016/j.jeconom.2007.05.001

11. Imbens GW, Rubin DB. Estimating outcome distributions for compliers in instrumental variables models. Rev Econ Stud. 1997;64: 555–574. doi:10.2307/2971731

12. Abadie A. Bootstrap tests for distributional treatment effects in instrumental variable models. J Am Stat Assoc. 2002;97: 284–292. doi:10.1198/016214502753479419

**Appendix C: Supplementary Figures**

**Fig C1. Sensitivity analysis: retention in care 12-months (lab results only)**

Notes: 12-month retention is defined as having a CD4 count, viral load, or initiating ART within the period 6-18 months. Sample excludes patients with <18 months potential follow-up. Local linear regression estimated with IK optimal bandwidth = 116.8 cells.

**Fig C2. Percent retained in care at 0-6, 6-12, 12-18, and 18-24 months (lab-results only)**

Note: Retention is defined as having a laboratory test or initiating ART within the 6-month interval. Local linear regression estimated with Imbens-Kalyanaraman (IK) optimal bandwidths of 114.2 (0-6mo), 164.7 (6-12mo), 125.4 (12-18mo), 164.2 (18-24mo).

**Fig C3. Proportion starting ART within 2 weeks, 6 months, and 12 months**

Notes: Having an eligible CD4 count (<350 cells) had no effect on initiation rates in the first two weeks, when patients would only have been initiated if they were very sick. An eligible CD4 count led to a large increase in probability of ART initiation between 2 weeks and six months. Between six and twelve months, there was little difference in the number of new initiators by baseline eligibility.

**Appendix D: Supplementary Tables**

**Table D1. Intent-to-treat effects of ART eligibility on ART initiation and retention in care: Imbens-Kalyanaraman bandwidth**

*Risk difference*100 (95% CI); P value*

|  | (1) | (2) | (3) | (4) | (5) | (6) | (7) |
| --- | --- | --- | --- | --- | --- | --- | --- |
| Outcomes: | ART 6mo | Retained  at 12mo | Retained  0-6mo | Retained  6-12mo | Retained  12-18mo | Retained  18-24mo | Retained  at 12mo (alt) |
|  |  |  |  |  |  |  |  |
| First CD4 < 350 cells | 25.4 | 17.9 | 17.1 | 8.2 | 4.6 | 9.1 | 11.2 |
|  | (19.7, 31.1) | (11.4, 24.3) | (11.3, 22.9) | (3.8, 12.6) | (-1.0, 10.1) | (2.4, 15.8) | (4.2, 18.1) |
|  | <0.001 | <0.001 | <0.001 | <0.001 | 0.108 | 0.007 | 0.002 |
|  |  |  |  |  |  |  |  |
| Value of first CD4 | -0.14 | 0.00 | -0.11 | -0.02 | 0.02 | 0.06 | -0.01 |
|  | (-0.20, -0.08) | (-0.06, 0.06) | (-0.17, -0.05) | (-0.05, 0.02) | (-0.04, 0.08) | (0.00, 0.11) | (-0.09, 0.07) |
|  | <0.001 | 0.962 | 0.001 | 0.364 | 0.536 | 0.035 | 0.798 |
|  |  |  |  |  |  |  |  |
| First CD4 < 350 cells  * Value of first CD4 | 0.11 | -0.01 | 0.07 | -0.01 | -0.03 | -0.07 | -0.02 |
|  | (0.01, 0.21) | (-0.09, 0.07) | (-0.02, 0.16) | (-0.06, 0.04) | (-0.11, 0.05) | (-0.15, 0.00) | (-0.12, 0.09) |
|  | 0.033 | 0.806 | 0.130 | 0.627 | 0.503 | 0.063 | 0.739 |
|  |  |  |  |  |  |  |  |
| Constant | 17.8 | 31.8 | 30.3 | 20.6 | 17.2 | 9.9 | 29.9 |
|  | (14.3, 21.4) | (27.3, 36.4) | (26.3, 34.3) | (17.6, 23.6) | (13.2, 21.1) | (5.4, 14.4) | (25.0, 34.8) |
|  | <0.001 | <0.001 | <0.001 | <0.001 | <0.001 | <0.001 | <0.001 |
|  |  |  |  |  |  |  |  |
| IK bw, cells | 96.4 | 142.1 | 114.2 | 164.7 | 125.4 | 164.2 | 116.8 |
| N | 3,354 | 3,327 | 3,937 | 5,478 | 2,954 | 1,734 | 2,733 |
|  |  |  |  |  |  |  |  |
| Notes: Each column is a separate linear probability regression model. Results are presented on a percentage point scale (x100). Each cell displays regression coefficient, heteroskedasticity-robust 95% CI, and P-value for the test that the coefficient is equal to zero. Models were estimated for a window of data around the threshold equal to twice the Imbens-Kalyanaraman (IK) optimal bandwidth, which was estimated separately (and is reported separately) for each outcome. The regressions reported here are the basis for the results reported in Table 2. | | | | | | | |

**Table D2. Intent-to-treat effects of ART eligibility on ART initiation and retention in care: bandwidth = 50 cells.**

*Risk difference*100 (95% CI); P value*

|  | (1) | (2) | (3) | (4) | (5) | (6) | (7) |  |
| --- | --- | --- | --- | --- | --- | --- | --- | --- |
| Outcomes: | ART 6mo | Retained at 12mo | Retained 0-6mo | Retained 6-12mo | Retained 12-18mo | Retained 18-24mo | Retained 12mo (alt) |  |
|  |  |  |  |  |  |  |  |  |
| First CD4 < 350 cells | 19.03 | 10.06 | 13.88 | 7.55 | 2.99 | 8.64 | 7.90 |  |
|  | (10.95, 27.11) | (-0.61, 20.7) | (5.18, 22.58) | (-0.22, 15.32) | (-5.62, 11.60) | (-2.98, 20.26) | (-2.52, 18.33) |  |
|  | <0.001 | 0.064 | 0.002 | 0.057 | 0.496 | 0.145 | 0.137 |  |
|  |  |  |  |  |  |  |  |  |
| Value of first CD4 | -0.37 | -0.18 | -0.22 | -0.03 | 0.02 | 0.14 | -0.14 |  |
|  | (-0.54, -0.20) | (-0.44, 0.07) | (-0.43, -0.02) | (-0.21, 0.16) | (-0.19, 0.24) | (-0.17, 0.44) | (-0.39, 0.11) |  |
|  | <0.001 | 0.162 | 0.034 | 0.768 | 0.850 | 0.375 | 0.266 |  |
|  |  |  |  |  |  |  |  |  |
| First CD4 < 350 cells * Value of first CD4 | 0.29 | 0.05 | 0.17 | -0.00 | -0.05 | -0.17 | 0.11 |  |
|  | (0.01, 0.56) | (-0.33, 0.43) | (-0.13, 0.48) | (-0.28, 0.27) | (-0.36, 0.25) | (-0.60, 0.26) | (-0.26, 0.48) |  |
|  | 0.040 | 0.791 | 0.264 | 0.995 | 0.732 | 0.438 | 0.555 |  |
|  |  |  |  |  |  |  |  |  |
| Constant | 22.83 | 36.63 | 32.96 | 21.03 | 17.59 | 9.11 | 33.10 |  |
|  | (17.63, 28.03) | (29.08, 44.17) | (26.92, 39.01) | (15.72, 26.34) | (11.48, 23.71) | (1.03, 17.19) | (25.76, 40.45) |  |
|  | <0.001 | <0.001 | <0.001 | <0.001 | <0.001 | 0.027 | <0.001 |  |
|  |  |  |  |  |  |  |  |  |
| N | 1,806 | 1,234 | 1,806 | 1,806 | 1,234 | 550 | 1,234 |  |
| R^2^ | 0.12 | 0.03 | 0.05 | 0.01 | 0.00 | 0.01 | 0.02 |  |
|  |  |  |  |  |  |  |  |  |
| Notes: Each column is a separate linear probability regression model. Results are presented on a percentage point scale (x100). Each cell displays regression coefficient, 95% CI, and P-value for the test that the coefficient is equal to zero. Models were estimated for a window of data around the threshold equal to twice the bandwidth of 50 cells. Retained at 12mo includes all CD4 counts, VL, dates of initiation, and routine clinic visits; retention in the six-month intervals and retained 12m (alt) excludes routine clinic visits. | | | | | | | | |

**Table D3. Intent-to-treat effects of ART eligibility on ART initiation and retention in care: bandwidth = 100 cells.**

*Risk difference*100 (95% CI); P value*

|  | (1) | (2) | (3) | (4) | (5) | (6) | (7) |
| --- | --- | --- | --- | --- | --- | --- | --- |
| Outcomes: | ART 6mo | Retained at 12mo | Retained 0-6mo | Retained 6-12mo | Retained 12-18mo | Retained 18-24mo | Retained 12mo (alt) |
|  |  |  |  |  |  |  |  |
| First CD4 < 350 cells | 24.51 | 15.24 | 15.73 | 7.76 | 3.66 | 7.65 | 10.15 |
|  | (18.90, 30.13) | (7.63, 22.87) | (9.54, 21.91) | (2.18, 13.35) | (-2.59, 9.92) | (-0.69, 15.99) | (2.67, 17.64) |
|  | <0.001 | <0.001 | <0.001 | 0.006 | 0.251 | 0.072 | 0.008 |
|  |  |  |  |  |  |  |  |
| Value of first CD4 | -0.15 | -0.04 | -0.12 | -0.01 | 0.02 | 0.04 | -0.02 |
|  | (-0.20, -0.09) | (-0.14, 0.06) | (-0.20, -0.04) | (-0.08, 0.06) | (-0.06, 0.10) | (-0.06, 0.14) | (-0.12, 0.07) |
|  | <0.001 | 0.424 | 0.002 | 0.754 | 0.601 | 0.451 | 0.618 |
|  |  |  |  |  |  |  |  |
| First CD4 < 350 cells * Value of first CD4 | 0.09 | 0.00 | 0.05 | -0.03 | -0.06 | -0.07 | -0.02 |
|  | (-0.01, 0.19) | (-0.13, 0.14) | (-0.06, 0.16) | (-0.13, 0.07) | (-0.17, 0.05) | (-0.22, 0.08) | (-0.15, 0.11) |
|  | 0.066 | 0.990 | 0.353 | 0.523 | 0.295 | 0.340 | 0.771 |
|  |  |  |  |  |  |  |  |
| Constant | 17.91 | 33.35 | 30.70 | 20.53 | 16.93 | 10.44 | 30.28 |
|  | (14.46, 21.36) | (27.95, 38.75) | (26.43, 34.98) | (16.71, 24.36) | (12.49, 21.36) | (4.81, 16.06) | (24.99, 35.56) |
|  | <0.001 | <0.001 | <0.001 | <0.001 | <0.001 | <0.001 | <0.001 |
|  |  |  |  |  |  |  |  |
| N | 3,460 | 2,366 | 3,460 | 3,460 | 2,366 | 1,101 | 2,366 |
| R^2^ | 0.14 | 0.04 | 0.07 | 0.01 | 0.00 | 0.01 | 0.02 |
|  |  |  |  |  |  |  |  |
| Notes: Each column is a separate linear probability regression model. Results are presented on a percentage point scale (x100). Each cell displays regression coefficient, 95% CI, and P-value for the test that the coefficient is equal to zero. Models were estimated for a window of data around the threshold equal to twice the bandwidth of 100 cells. Retained at 12mo includes all CD4 counts, VL, dates of initiation, and routine clinic visits; retention in the six-month intervals and retained 12m (alt) excludes routine clinic visits. | | | | | | | |

**Table D4. Intent-to-treat effects of ART eligibility on ART initiation and retention in care: bandwidth = 150 cells.**

*Risk difference*100 (95% CI); P value*

|  | (1) | (2) | (3) | (4) | (5) | (6) | (7) |
| --- | --- | --- | --- | --- | --- | --- | --- |
| Outcomes: | ART 6mo | Retained at 12mo | Retained 0-6mo | Retained 6-12mo | Retained 12-18mo | Retained 18-24mo | Retained 12mo (alt) |
|  |  |  |  |  |  |  |  |
| First CD4 < 350 cells | 27.38 | 17.79 | 18.64 | 7.99 | 5.06 | 8.82 | 12.59 |
|  | (22.83, 31.92) | (11.52, 24.06) | (13.58, 23.70) | (3.40, 12.57) | (-0.10, 10.21) | (1.82, 15.82) | (6.44, 18.75) |
|  | <0.001 | <0.001 | <0.001 | 0.001 | 0.055 | 0.014 | <0.001 |
|  |  |  |  |  |  |  |  |
| Value of first CD4 | -0.07 | 0.01 | -0.06 | -0.02 | 0.04 | 0.08 | 0.02 |
|  | (-0.10, -0.04) | (-0.04, 0.07) | (-0.10, -0.02) | (-0.06, 0.02) | (-0.01, 0.08) | (0.02, 0.14) | (-0.03, 0.07) |
|  | <0.001 | 0.669 | 0.003 | 0.315 | 0.130 | 0.012 | 0.458 |
|  |  |  |  |  |  |  |  |
| First CD4 < 350 cells  * Value of first CD4 | 0.02 | -0.03 | 0.01 | -0.01 | -0.05 | -0.12 | -0.04 |
|  | (-0.03, 0.07) | (-0.11, 0.04) | (-0.05, 0.07) | (-0.06, 0.05) | (-0.11, 0.01) | (-0.21, -0.03) | (-0.12, 0.03) |
|  | 0.409 | 0.405 | 0.666 | 0.753 | 0.128 | 0.008 | 0.236 |
|  |  |  |  |  |  |  |  |
| Constant | 15.16 | 31.37 | 28.51 | 20.78 | 16.41 | 8.83 | 28.55 |
|  | (12.44, 17.89) | (26.91, 35.83) | (25.01, 32.00) | (17.64, 23.92) | (12.75, 20.07) | (4.10, 13.57) | (24.19, 32.91) |
|  | <0.001 | <0.001 | <0.001 | <0.001 | <0.001 | <0.001 | <0.001 |
|  |  |  |  |  |  |  |  |
| N | 5,066 | 3,490 | 5,066 | 5,066 | 3,490 | 1,613 | 3,490 |
| R^2^ | 0.15 | 0.03 | 0.08 | 0.02 | 0.00 | 0.01 | 0.02 |
|  |  |  |  |  |  |  |  |
| Notes: Each column is a separate linear probability regression model. Results are presented on a percentage point scale (x100). Each cell displays regression coefficient, 95% CI, and P-value for the test that the coefficient is equal to zero. Models were estimated for a window of data around the threshold equal to twice the bandwidth of 150 cells. Retained at 12mo includes all CD4 counts, VL, dates of initiation, and routine clinic visits; retention in the six-month intervals and retained 12m (alt) excludes routine clinic visits. | | | | | | | |

**Table D5. Intent-to-treat effects of ART eligibility on ART initiation and retention in care: bandwidth = 200 cells.**

*Risk difference*100 (95% CI); P value*

|  | (1) | (2) | (3) | (4) | (5) | (6) | (7) |
| --- | --- | --- | --- | --- | --- | --- | --- |
| Outcomes: | ART 6mo | Retained at 12mo | Retained 0-6mo | Retained 6-12mo | Retained 12-18mo | Retained 18-24mo | Retained 12mo (alt) |
|  |  |  |  |  |  |  |  |
| First CD4 < 350 cells | 28.12 | 16.91 | 18.53 | 8.65 | 4.47 | 7.79 | 12.56 |
|  | (24.18, 32.07) | (11.39, 22.43) | (14.12, 22.93) | (4.62, 12.68) | (-0.07, 9.02) | (1.66, 13.91) | (7.13, 17.99) |
|  | <0.001 | <0.001 | <0.001 | <0.001 | 0.054 | 0.013 | <0.001 |
|  |  |  |  |  |  |  |  |
| Value of first CD4 | -0.05 | 0.01 | -0.06 | -0.01 | 0.02 | 0.04 | 0.02 |
|  | (-0.07, -0.03) | (-0.03, 0.05) | (-0.09, -0.03) | (-0.04, 0.01) | (-0.01, 0.05) | (-0.00, 0.08) | (-0.02, 0.06) |
|  | <0.001 | 0.585 | <0.001 | 0.332 | 0.195 | 0.077 | 0.281 |
|  |  |  |  |  |  |  |  |
| First CD4 < 350 cells  * Value of first CD4 | -0.01 | -0.04 | 0.00 | -0.01 | -0.03 | -0.05 | -0.04 |
|  | (-0.05, 0.02) | (-0.09, 0.01) | (-0.04, 0.04) | (-0.05, 0.03) | (-0.07, 0.01) | (-0.11, 0.00) | (-0.09, 0.01) |
|  | 0.512 | 0.092 | 0.985 | 0.608 | 0.182 | 0.065 | 0.084 |
|  |  |  |  |  |  |  |  |
| Constant | 13.93 | 31.48 | 28.25 | 20.45 | 17.24 | 10.99 | 28.60 |
|  | (11.58, 16.27) | (27.54, 35.43) | (25.21, 31.30) | (17.67, 23.22) | (14.01, 20.47) | (6.85, 15.14) | (24.74, 32.46) |
|  | <0.001 | <0.001 | <0.001 | <0.001 | <0.001 | <0.001 | <0.001 |
|  |  |  |  |  |  |  |  |
| N | 6,460 | 4,420 | 6,460 | 6,460 | 4,420 | 2,030 | 4,420 |
| R^2^ | 0.17 | 0.04 | 0.09 | 0.02 | 0.00 | 0.01 | 0.02 |
|  |  |  |  |  |  |  |  |
| Notes: Each column is a separate linear probability regression model. Results are presented on a percentage point scale (x100). Each cell displays regression coefficient, 95% CI, and P-value for the test that the coefficient is equal to zero. Models were estimated for a window of data around the threshold equal to twice the bandwidth of 200 cells. Retained at 12mo includes all CD4 counts, VL, dates of initiation, and routine clinic visits; retention in the six-month intervals and retained 12m (alt) excludes routine clinic visits. | | | | | | | |

**Table D6. Comparison of local linear and local logistic regression estimates: Intent-to-treat effects of ART eligibility on ART initiation and retention in care**

*Risk difference*100 (95% CI); P value*

|  | (1) | (2) | (3) | (4) | (5) | (6) | (7) |
| --- | --- | --- | --- | --- | --- | --- | --- |
| Outcomes: | ART 6mo | Retained  at 12mo | Retained  0-6mo | Retained  6-12mo | Retained  12-18mo | Retained  18-24mo | Retained at 12mo (alt) |
|  |  |  |  |  |  |  |  |
| Effect estimate,  local linear model (same as Table 2) | 25.3 | 17.9 | 17.1 | 8.2 | 4.6 | 9.1 | 11.2 |
|  | (19.7, 31.1) | (11.4, 23.3) | (11.3, 22.9) | (3.8, 12.6) | (-1.0, 10.1) | (2.5, 15.8) | (4.2, 18.1) |
|  | <0.001 | <0.001 | <0.001 | <0.001 | 0.108 | 0.007 | 0.002 |
|  |  |  |  |  |  |  |  |
| Effect estimate,  local logistic model | 24.1 | 17.9 | 16.8 | 8.2 | 4.5 | 8.7 | 11.2 |
|  | (17.9, 30.5) | (11.5, 24.3) | (10.8, 22.7) | (3.8, 12.6) | (-0.9, 10.0) | (2.6, 14.9) | (4.2, 18.1) |
|  | <0.001 | <0.001 | <0.001 | <0.001 | 0.103 | 0.006 | 0.002 |
|  |  |  |  |  |  |  |  |
| IK bw, cells | 96.4 | 142.1 | 114.2 | 164.7 | 125.4 | 164.2 | 116.8 |
| N | 3,354 | 3,327 | 3,937 | 5,478 | 2,954 | 1,734 | 2,733 |
|  |  |  |  |  |  |  |  |
| Notes: Local linear effect estimates are replayed from Table 2. Local logistic regression estimates are the difference in predicted probabilities at the threshold, obtained after running a logistic regression model using the same bandwidth. Results are presented on a percentage point scale (x100). Each cell displays the effect estimate, 95% CI, and P-value for the test that the effect estimate is equal to zero. Retained at 12mo includes all CD4 counts, VL, dates of initiation, and routine clinic visits; retention in the six-month intervals and retained 12m (alt) excludes routine clinic visits. | | | | | | | |

**Table D7. Conditional on treated and conditional on control regression discontinuity models. (Estimated for the purpose of calculating treated and control complier means). Bandwidth: 100 cells.**

*Risk difference*100 (95% CI); P value*

|  | (1) | (2) | (3) | (4) |
| --- | --- | --- | --- | --- |
| Outcomes: | ART 6mo | Retained at 12mo | Retained at 12mo | Retained at 12mo |
| Sample: | *Full* | *Full* | *Controls* | *Treated* |
|  |  |  |  |  |
| First CD4 < 350 cells | 21.72 | 15.25 | 0.45 | 2.22 |
|  | (14.93, 28.51) | (7.63, 22.87) | (-7.32, 8.23) | (-8.59, 13.02) |
|  | <0.001 | <0.001 | 0.909 | 0.687 |
|  |  |  |  |  |
| Value of first CD4 | -0.12 | -0.04 | 0.04 | 0.04 |
|  | (-0.20, -0.05) | (-0.14, 0.06) | (-0.06, 0.14) | (-0.15, 0.23) |
|  | 0.001 | 0.424 | 0.408 | 0.667 |
|  |  |  |  |  |
| First CD4 < 350 cells  * Value of first CD4 | 0.02 | 0.00 | 0.01 | -0.03 |
|  | (-0.10, 0.14) | (-0.13, 0.14) | (-0.13, 0.15) | (-0.24, 0.18) |
|  | 0.723 | 0.990 | 0.883 | 0.762 |
|  |  |  |  |  |
| Constant | 17.98 | 33.35 | 21.61 | 86.91 |
|  | (13.75, 22.20) | (27.95, 38.75) | (16.34, 26.88) | (77.55, 96.27) |
|  | <0.001 | <0.001 | <0.001 | <0.001 |
|  |  |  |  |  |
| N | 2,366 | 2,366 | 1,653 | 713 |
| R^2^ | 0.13 | 0.04 | 0.00 | 0.00 |
|  |  |  |  |  |
| Notes: Each column is a separate linear probability regression model. Retained at 12mo includes all CD4 counts, VL, dates of initiation, and routine clinic visits. The treated sample are patients who initiated ART within the first six months after their earliest CD4 count; the controls are patients who did not initiate ART within the first six months. Results are presented on a percentage point scale (x100). Each cell displays regression coefficient, 95% CI, and P-value for the test that the coefficient is equal to zero. Models were estimated for a window of data around the threshold equal to twice the bandwidth of 100 cells. | | | | |
