# Supplementary material for: Treatment eligibility and retention in clinical HIV care: A regression discontinuity study in South Africa
Source: PLoS Med. 2017 Nov 28;14(11):e1002463. doi: 10.1371/journal.pmed.1002463 (PMC5705070; doi:10.1371/journal.pmed.1002463)
Supplement: S1 Data — (DOCX) [file pmed.1002463.s003.docx]

S1_Data

This analysis utilized data from the ARTemis data system, which was a collaboration between the Africa Health Research Institute ( AHRI, [www.ahri.org](http://www.ahri.org)) and Provincial Department of Health. ARTemis included all patients who sought care in the Hlabisa (sub-district) HIV Treatment and Care Programme during the study period. These data also known as the Hlabisa Cohort have been used extensively in publications by researchers at AHRI (formerly the Africa Centre for Population Health) as well as by researchers affiliated with the IeDEA Collaboration (www.iedea.org), of which AHRI is a contributing member.

Background information on the Hlabisa HIV Treatment and Care Programme can be found in the Cohort Profile published by Houlihan and colleagues (2011) in the International Journal of Epidemiology. <https://www.ncbi.nlm.nih.gov/pmc/articles/PMC3195268/>

The raw data and data documentation used in this analysis can be obtained from the AHRI data repository: <https://data.africacentre.ac.za>.

The datasets required for the analysis are:

- “AH03-01 All patients in HIV Care.dta"
- “AH03-02 All Lab Tests.dta"
- “AH03-03 ART Clinic Visits.dta"

Code to clean and analyze these datasets are contained in S1_Code.
