# Supplementary material for: Treatment eligibility and retention in clinical HIV care: A regression discontinuity study in South Africa
Source: PLoS Med. 2017 Nov 28;14(11):e1002463. doi: 10.1371/journal.pmed.1002463 (PMC5705070; doi:10.1371/journal.pmed.1002463)
Supplement: S1 STROBE Checklist — (DOC) [file pmed.1002463.s004.doc]

STROBE Statement—Checklist of items that should be included in reports of ***cohort studies***

|  | Item No | Recommendation |
| --- | --- | --- |
| **Title and abstract** | 1 | (*a*) Indicate the study’s design with a commonly used term in the title or the abstract  *Treatment eligibility and retention in clinical HIV care: regression-discontinuity evidence from South Africa* |
|  |  | (*b*) Provide in the abstract an informative and balanced summary of what was done and what was found  *See text of the Abstract.* |
| Introduction | | |
| Background/rationale | 2 | Explain the scientific background and rationale for the investigation being reported  *Introduction, paragraphs 1-2* |
| Objectives | 3 | State specific objectives, including any prespecified hypotheses  *Introduction, paragraph 3* |
| Methods | | |
| Study design | 4 | Present key elements of study design early in the paper  *Introduction, paragraph 3* |
| Setting | 5 | Describe the setting, locations, and relevant dates, including periods of recruitment, exposure, follow-up, and data collection  *Methods, paragraphs 2-3* |
| Participants | 6 | (*a*) Give the eligibility criteria, and the sources and methods of selection of participants. Describe methods of follow-up  *Methods, paragraphs 2-3* |
|  |  | (*b*) For matched studies, give matching criteria and number of exposed and unexposed  *N/A* |
| Variables | 7 | Clearly define all outcomes, exposures, predictors, potential confounders, and effect modifiers. Give diagnostic criteria, if applicable  *Methods, paragraphs 4-8* |
| Data sources/ measurement | 8* | For each variable of interest, give sources of data and details of methods of assessment (measurement). Describe comparability of assessment methods if there is more than one group  *Methods, paragraphs 4-8* |
| Bias | 9 | Describe any efforts to address potential sources of bias  *Methods, paragraphs 9-12* |
| Study size | 10 | Explain how the study size was arrived at  *We used all available data. Methods, paragraphs 2-3.* |
| Quantitative variables | 11 | Explain how quantitative variables were handled in the analyses. If applicable, describe which groupings were chosen and why  *Methods, paragraphs 4-8* |
| Statistical methods | 12 | (*a*) Describe all statistical methods, including those used to control for confounding  *Methods, paragraphs 9-12* |
|  |  | (*b*) Describe any methods used to examine subgroups and interactions  *No subgroup analysis was conducted.* |
|  |  | (*c*) Explain how missing data were addressed  *There were no known missing data.* |
|  |  | (*d*) If applicable, explain how loss to follow-up was addressed  *Loss to follow-up was the outcome of interest.*  *Methods, paragraphs 4-8* |
|  |  | (*e*) Describe any sensitivity analyses  *Methods, paragraph 5: Alternate definition of outcome.*  *Methods, paragraph 10: Robustness to alternate bandwidths and using logistic regression instead of a linear probability model* |
| Results | | |
| Participants | 13* | (a) Report numbers of individuals at each stage of study—eg numbers potentially eligible, examined for eligibility, confirmed eligible, included in the study, completing follow-up, and analysed  *Results, paragraph 1* |
|  |  | (b) Give reasons for non-participation at each stage  *There was no non-participation as the study was an analysis of clinical records.* |
|  |  | (c) Consider use of a flow diagram  We decided against a flow diagram given that participants were not recruited for the study – it was an analysis of existing clinical records – and the inclusion/exclusion criteria were very simple. |
| Descriptive data | 14* | (a) Give characteristics of study participants (eg demographic, clinical, social) and information on exposures and potential confounders  *Results, paragraph 1* |
|  |  | (b) Indicate number of participants with missing data for each variable of interest  *There was no missing data.* |
|  |  | (c) Summarise follow-up time (eg, average and total amount)  *The analysis was not conducted using person-time. All individuals were followed for at least 12 months.* |
| Outcome data | 15* | Report numbers of outcome events or summary measures over time  *Results, paragraph 2* |
| Main results | 16 | (*a*) Give unadjusted estimates and, if applicable, confounder-adjusted estimates and their precision (eg, 95% confidence interval). Make clear which confounders were adjusted for and why they were included  *Results, paragraph 2. No confounders were adjusted for as it is unnecessary in this quasi-experimental design (similar to an RCT). Table 1 showed that there was balance between exposed/unexposed in observed baseline characteristics.* |
|  |  | (*b*) Report category boundaries when continuous variables were categorized  *Methods, paragraph 5: “As secondary outcomes, we assessed the presence of a CD4, viral load, or ART start date within six-month intervals following a patient’s first CD4 count, out to two years (0-6, 6-12, 12-18, 18-24 months).”* |
|  |  | (*c*) If relevant, consider translating estimates of relative risk into absolute risk for a meaningful time period  *All estimates were reported as absolute risk differences.* |
| Other analyses | 17 | Report other analyses done—eg analyses of subgroups and interactions, and sensitivity analyses  *Results, paragraphs 3-4* |
| Discussion | | |
| Key results | 18 | Summarise key results with reference to study objectives  *Discussion, paragraphs 1-6* |
| Limitations | 19 | Discuss limitations of the study, taking into account sources of potential bias or imprecision. Discuss both direction and magnitude of any potential bias  *Discussion, paragraphs 13-16* |
| Interpretation | 20 | Give a cautious overall interpretation of results considering objectives, limitations, multiplicity of analyses, results from similar studies, and other relevant evidence  *Discussion, paragraphs 17-20* |
| Generalisability | 21 | Discuss the generalisability (external validity) of the study results  *Discussion, paragraph 14* |
| Other information | | |
| Funding | 22 | Give the source of funding and the role of the funders for the present study and, if applicable, for the original study on which the present article is based  *See funding acknowledgments.* |

*Give information separately for exposed and unexposed groups.

**Note:** An Explanation and Elaboration article discusses each checklist item and gives methodological background and published examples of transparent reporting. The STROBE checklist is best used in conjunction with this article (freely available on the Web sites of PLoS Medicine at http://www.plosmedicine.org/, Annals of Internal Medicine at http://www.annals.org/, and Epidemiology at http://www.epidem.com/). Information on the STROBE Initiative is available at http://www.strobe-statement.org.
